# Population density and timing of breeding mediate effects of early life conditions on recruitment

**DOI:** 10.1098/rsbl.2024.0689

**Published:** 2025-04-23

**Authors:** Sarah D. Mueller, Nathaniel T. Wheelwright, Daniel J. Mennill, Amy E. M. Newman, Stéphanie M. Doucet, Joseph B. Burant, Sarah L. Dobney, Greg W. Mitchell, Hayley A. Spina, D. Ryan Norris

**Affiliations:** ^1^ Department of Integrative Biology, Guelph University, Guelph, Ontario, Canada; ^2^ Department of Biology, Bowdoin College, Brunswick, ME, USA; ^3^ Department of Integrative Biology, University of Windsor, Windsor, Ontario, Canada; ^4^ Biological Sciences, University of Windsor, Windsor, Ontario, Canada; ^5^ Department of Animal Ecology, Netherlands Institute of Ecology, Wageningen, The Netherlands; ^6^ Wildlife Research Division, Environment and Climate Change Canada, Ottawa, Ontario, Canada

**Keywords:** juvenile survival, Savannah sparrow, carry-over effects, reproduction, demography

## Abstract

Identifying the factors driving juvenile recruitment is crucial for predicting the response of populations to environmental change. Importantly, how early life conditions carry over to influence recruitment may be highly dependent on the context in which they occur. For example, the effects of challenging early life conditions may be more pronounced under high densities or when young are born late in the season. We examined the ecological factors influencing local recruitment spanning three decades in Savannah sparrows (*Passerculus sandwichensis*) breeding on Kent Island, NB, Canada. The effect of nestling mass on recruitment depended on both population density and fledging date. At low-population densities or early in the breeding season, nestling mass had little effect on recruitment probability. At high-population densities or later in the breeding season, mass had a stronger effect, with heavier individuals more likely to recruit. Lighter fledglings may have lower recruitment under challenging conditions due to lower competitive ability, lower mobility and greater susceptibility to resource limitation relative to heavier fledglings. Our findings have important implications for life-history evolution and selection on body size in a changing world, highlighting the relationships between population density, timing of breeding and offspring recruitment.

## 1. Introduction

Understanding the factors that drive survival and recruitment of juveniles is critical for predicting how wild populations will respond to environmental change [1–8]. Juvenile survival, which tends to be highly variable across years [2,5,9,10] and can be the dominant vital rate driving population abundance [2,4–6,8], is often influenced by conditions early in life [11–16]. Higher mass at birth, for example, results in enhanced competitive ability and better body condition after independence, leading to higher annual juvenile survival [3,11,16–22]. Large broods or litter sizes could result in more competition between young for parental care, reducing overall body condition in the nest and, consequently, juvenile survival [3] or could be an indicator of higher-quality parents and be associated with higher survival [3]. In all these cases, early life conditions can be strongly coupled to population dynamics via their effects on juvenile survival and, therefore, recruitment of young into the breeding population.

Importantly, the manner in which early life conditions carry over to influence survival and recruitment [23,24] may be highly dependent on the ecological context in which they occur [11,19,25,26]. These context-dependent effects can have important implications for not only understanding population dynamics but also the strength of selection over time. For example, population density during the breeding season can affect juvenile survival by increasing intraspecific competition for resources and predation risk [6,22]. To our knowledge, however, there is only one investigation of how density might interact with mass or condition of young: in a population of red deer (*Cervus elaphus*), over-winter survival of juveniles declined more strongly with decreasing birth mass when population density was high than when density was low [11]. Similarly, timing of breeding could shape the effects of early life conditions on survival. Early breeding typically improves survival of young because of higher early-season resource abundance, increased time for juveniles to build up body reserves and moult prior to migration or winter or increased dominance of early-born young [3,11,15,16,24,27–33]. Some studies have investigated whether later birth dates could pose additional challenges for individuals already experiencing poor early life conditions, such as low body mass or condition. In birds, nestling mass may influence survival of young in the first few weeks after leaving the nest among later-fledging individuals but not among earlier-fledging ones [34]. However, no studies of annual juvenile survival have found support for this prediction (no effect: [35,36]; opposite effect, i.e. lighter juveniles born later in the season had higher survival rates than heavier ones: [25]). Juvenile survival may depend on other interactions between early life traits and environmental conditions as well [26]. Opportunities to study the influence of complex interactions between early life conditions on recruitment such as these may be limited by small sample sizes and the challenge of obtaining a long enough time series to capture sufficient variation in annual conditions.

We examined the ecological factors influencing local recruitment spanning nearly 30 generations in a population of Savannah sparrows (*Passerculus sandwichensis*) breeding on Kent Island, NB, Canada. Juvenile survival is an important driver of population dynamics of Savannah sparrows on Kent Island [6]. Two previous studies on this population revealed that recruitment was not affected by the sex of the juvenile or year of birth but was higher for heavier juveniles and juveniles fledged from nests initiated early in the breeding season [37,38]. However, these studies were based on fewer years of observation, considered a smaller set of predictors and, most importantly, did not evaluate interactive effects between population density, mass and time of breeding. Here, we used 27 years of breeding and demographic data between 1987 and 2022 to examine how interactive effects between early life conditions and ecological context influenced local recruitment rates of fledglings. We hypothesized that the effect of mass on recruitment would depend on population density, because lighter juveniles may be more vulnerable to elevated levels of competition experienced at high densities, and on fledge date, because lighter juveniles may be less able to cope with lower late-season resource abundance and with reduced time to prepare for migration.

## 2. Methods

### (a) Study system and field methods

Savannah sparrows are migratory grassland songbirds that breed across North America and winter in Central America and southern North America [39]. Our study population breeds on Kent Island, NB, Canada (44.58° N, 66.75° W), a small island in the Bay of Fundy, and has been studied annually since 1987, excluding 2005−2007 and 2020. From May to July each year, we mapped all territories, monitored all nests and colour banded all nestlings and new members of the population within an approximately 10 ha study area (see [40] for detailed methods). All breeding adults were given a unique combination of a US Fish and Wildlife Service aluminium leg band and three coloured plastic leg bands. Ages of breeding adults, categorized as yearling (second-year (SY)) or older (after second-year (ASY)), were known with certainty if they were banded as juveniles in preceding years or were estimated using feather shape and wear if they were immigrants [41]. We calculated annual population density (birds ha^−1^) as the number of breeding adults on the main study site each year, divided by the area of the study site (10.7 ha) [40].

Nests were primarily found during incubation (90% of nests; 2% found during laying and 8% with nestlings) and, once found, were monitored every other day to determine clutch size (range 2–6 eggs), number of nestlings (range 1–6), hatching and fledge dates, and nest fate (failed or succeeded). Nestlings were banded, weighed and measured on day 7 after hatching, 2 days before expected fledging, to avoid inducing premature fledging due to nest disturbance. Nestlings were given one aluminium leg band and one coloured leg band. We assumed that all nestlings present during banding fledged if the nest was successful. If they returned the following year to breed, they were captured and given a full colour band combination as described above. Females can successfully fledge up to two broods per year (on average, approx. 30% of females attempt a second brood) [40]. The mating status of a female—as the mate of a monogamous male or as the primary (first-laying) or secondary (later-laying) mate of a polygynous male—was determined by assigning social partners using behaviours including territoriality, copulation, mate-guarding and nestling feeding (see [42]).

We defined ‘recruitment’ as the return rate between the year an individual was banded as a nestling and the following breeding season and assumed that individuals not resighted in any following year had died. Strong natal and breeding philopatry in this population make dispersal far outside the study area unlikely (median natal dispersal distance = 228 m, median breeding dispersal distance 17 m for females, 32 m for males) [43,44]. Additionally, each year, only 1–4 dispersed yearlings are found during censuses where two researchers walk transects and search for birds on Kent Island outside the study area and on the neighbouring islands (DR Norris, JB Burant, SD Mueller, unpublished data, 2024). Detection probability was high: only 35 birds in our recruitment dataset were not detected as yearlings but were detected in a subsequent year, out of 4870 individuals (0.7%). Therefore, we did not use capture–recapture analysis to estimate apparent survival.

### (b) Statistical analysis

Recruitment of young from fledging to their first breeding season, defined as whether a fledgling was recorded breeding in subsequent years, was modelled using logistic regression (GLMM, binomial distribution, logit link) with random intercepts for year, female identity and nest identity (nested within female identity). Unless otherwise noted, all continuous predictors were grand-mean centred, but not scaled, to improve interpretability and reduce multicollinearity. Models were fit using sum-to-zero coding for categorical predictors. The full model included the mating status of the fledgling’s mother (monogamous, primary polygynous or secondary polygynous), which can influence the amount of parental care received, population density, ages of the parents (both SY, one SY and one ASY, or both ASY), because older parents typically have higher breeding success in this population [45]; fledge date (group-mean centred by female status and brood number); brood number; mass as a nestling; and number of young that fledged from the nest. We also included interactions between female status and population density, female status and parent’s age, population density and parent’s age, population density and fledge date, population density and mass, and fledge date and mass. We then fit a final model after removing non-significant (*p* < 0.05) interaction terms so that we could interpret main effects, leaving us with only the interactions mass × population density and mass × fledge date. For the few juveniles missing fledge dates, we estimated fledge date by adding 9 days to the hatch day (mean difference between hatch and fledge day ± s.e.: 9.18 ± 0.01 days). We excluded individuals born in 2004 and 2019 because no monitoring took place in the subsequent year. Analyses were conducted using R (v. 4.4.0; [46]). Models were fit using glmmTMB (v. 1.1.9; [47]) and diagnostics checked using DHARMa (v. 0.4.6; [48]). Pairwise comparisons between levels of categorical variables were conducted using emmeans, adjusted for multiple comparisons using the Tukey method (v. 1.10.1; [49]).

## 3. Results

**Figure 1.**
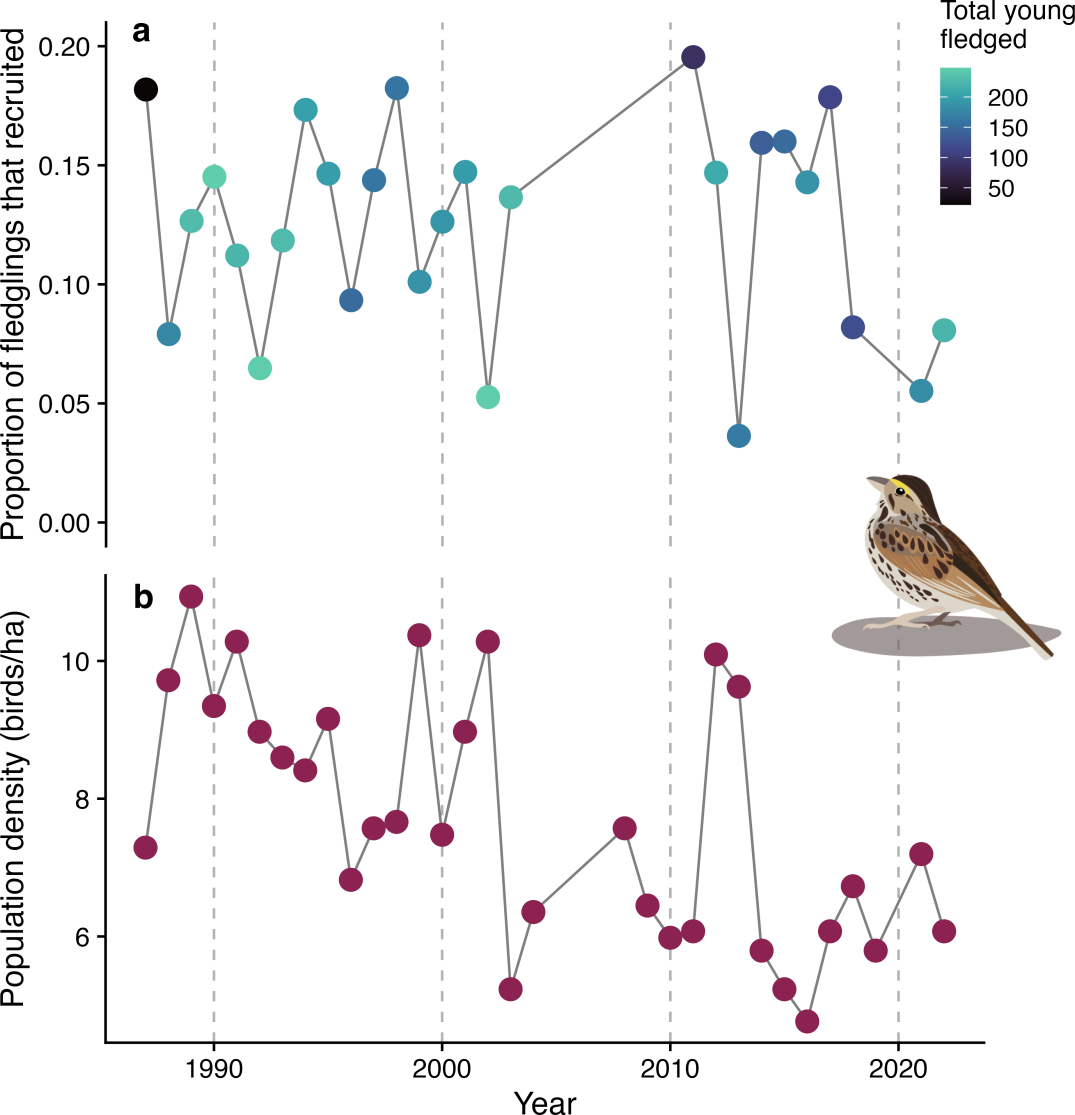
(a) Recruitment rate of fledgling Savannah sparrows (*Passerculus sandwichensis*) varied across years. On average, 12% of fledglings were recruited (range 0.04–0.19, *n* = 4870 individuals, *n* = 1364 nests, *n* = 27 years). (b) Population density (the number of breeding adults per hectare) varied across years. Savannah sparrow graphic by Shelby Bohn.

Across our 27 year study of context dependence of recruitment of juvenile Savannah sparrows, the yearly mean recruitment rate into the study population was 12% (range 0.04–0.19, *n* = 4870 individuals, 1364 nests, 27 years; figure 1a). The mean mass of nestlings was 14.6 g (range 4.4–20.5 g), and mean population density was 8.2 birds ha^−1^ (range 4.8–10.9 birds ha^−1^, figure 1b). On average, first brood young fledged on 30 June ± 11 days (s.d.) and second brood young fledged on 21 July ± 6 days.

**Figure 2.**
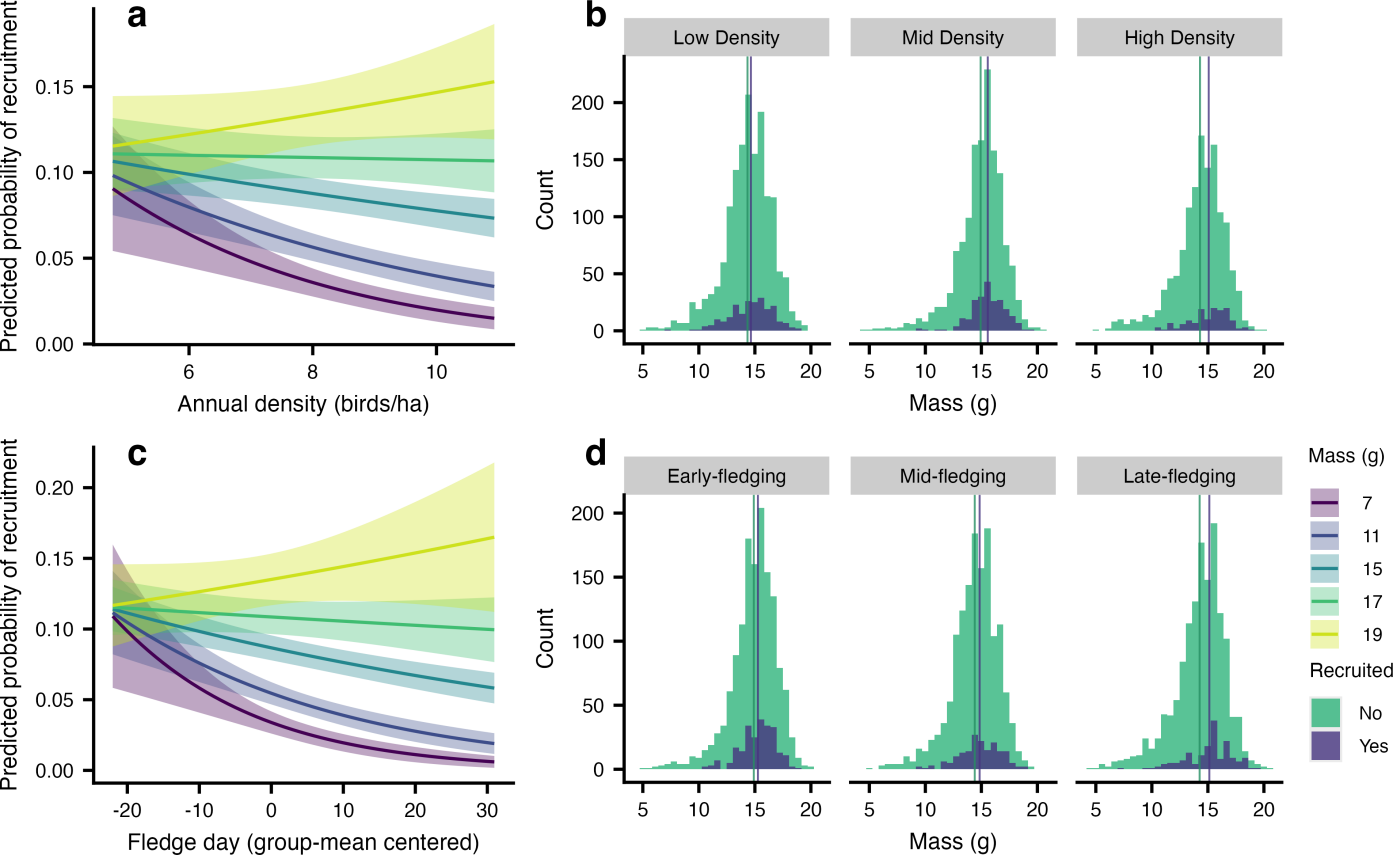
The effects of nestling mass on recruitment in Savannah sparrows (*Passerculus sandwichensis*) depended on both (a,b) population density (*p* = 0.04) and (c,d) fledging date (*p* = 0.03). (a) At low-population densities, recruitment probability did not differ by mass, but as population density increased, mass had an increasingly strong effect, with heavier fledglings more likely to recruit (GLMM; model predictions with s.e. generated using the R package emmeans, at mean values of other continuous predictors and averaged across levels of other categorical predictors). (b) Count of fledglings that were recruited or not by mass, binned by population density tertile. At lower densities, the distribution of fledglings that recruited versus those that did not overlapped more extensively than at higher densities, where recruits had higher mass than non-recruits. The difference in mean mass of fledglings that recruited versus those that did not, represented by vertical lines, was larger at high density. (c) For early-fledging juveniles, recruitment probability did not differ by mass, but among later-fledging juveniles, heavier individuals were more likely to recruit (GLMM; model predictions with s.e.). (d) Count of fledglings that were recruited or not by mass, binned by fledge date tertile. For early fledge dates, the distribution of fledglings that recruited versus those that did not overlapped more extensively than at later fledge dates, where recruits had higher mass than non-recruits. The difference in mean mass of fledglings that recruited versus those that did not, represented by vertical lines, was larger for later fledglings.

Nestling mass influenced recruitment, and this effect depended on both population density (GLMM, *p* = 0.04, figure 2, table 1) and fledging date (*p* = 0.03, figure 2, table 1). At low-population densities, recruitment probability did not differ by nestling mass, but as population density increased, nestling mass had an increasingly strong effect, with heavier individuals more likely to recruit (figure 2). Similarly, for juveniles fledging early in the season, recruitment probability did not differ by nestling mass, but for juveniles fledging later in the season, heavier individuals were more likely to recruit (figure 2). Fledglings from first broods were more likely to recruit than fledglings from second broods (first: 0.11 ± 0.01, *n* = 3838 birds; second: 0.06 ± 0.01, *n* = 1032 birds; *p* < 0.001, table 1). The mating status of the mother had a marginally significant effect on recruitment probability (type III ANOVA, χ^2^ = 4.68, d.f. = 2, *p* = 0.10), with offspring of secondary polygynous females somewhat less likely to recruit (probability ± s.e.: 0.07 ± 0.01, *n* = 726 birds) than offspring of primary polygynous females (0.10 ± 0.01; pairwise comparison, *p* = 0.08, *n* = 824 birds). Recruitment probability of offspring of monogamous females did not differ from that of offspring of primary or secondary polygynous females (monogamous: 0.08 ± 0.01, *n* = 3320 birds; pairwise comparisons, *p* > 0.20). Parents’ ages also had a marginally significant effect on recruitment probability (type III ANOVA table, χ^2^ = 4.91, d.f. = 2, *p* = 0.09), with offspring of two young parents (0.07 ± 0.01, *n* = 1346 birds) somewhat less likely to recruit than offspring of two older parents (0.10 ± 0.01, *n* = 1847 birds; pairwise comparison, *p* = 0.10). There was no significant difference in recruitment probability between offspring of mixed-age pairs (one older and one younger parent, 0.08 ± 0.01, *n* = 1677 birds) and offspring of either two young or two older parents (pairwise comparisons, *p* > 0.24).

**Table 1.** The effects of the mating status of mothers, population density, parents’ ages, fledge day, brood number, mass and the number of fledglings from the same nest on recruitment probability of juvenile Savannah sparrows (*Passerculus sandwichensis*). Parameter estimates for factor variables reflect sum-to-zero coding. The intercept is the grand mean across all levels of the factor and the estimate for each level is the deviation from the grand mean for that level. Estimates are on the logit scale.

fixed effects	estimate	s.e.	*z*-value	*p*‐value
(intercept)	−2.29	0.27	−8.64	<0.001
mating status (monogamous)	0.03	0.08	0.35	0.728
mating status (primary polygynous)	0.18	0.09	1.97	0.049
population density	−0.08	0.04	−1.87	0.061
parents’ ages (both older)	0.15	0.07	2.19	0.028
parents’ ages (one yearling, one older)	−0.04	0.07	−0.54	0.590
fledge day	−0.02	0.01	−3.02	0.003
brood number (1)	0.28	0.07	3.99	<0.001
mass	0.12	0.03	4.65	<0.001
number of fledglings	−0.03	0.07	−0.44	0.660
population density × mass	0.03	0.01	2.08	0.038
fledge day × mass	0.01	0.00	2.12	0.034
random effects	variance	s.d.		
year	0.08	0.29		
female ID	0.04	0.20		
nest ID	0.30	0.55		
*n* = 4870 fledglings, 693 females, 1364 nests, 27 years				

## 4. Discussion

In our 27 year study of recruitment in a wild population of Savannah sparrows, we found that recruitment was positively influenced by nestling mass and that this relationship depended on both population density and timing of breeding. The negative effect of low mass on recruitment when population density is high may be due to increased intraspecific competition leading to more agonistic interactions and resource limitation [6]. Lighter fledglings are less able to cope with these challenges due to lower competitive ability, lower mobility and greater susceptibility to resource limitation relative to heavier fledglings [19]. A similar result was found in red deer, where juvenile survival declined more strongly with decreasing mass at high density than at low density [11]. However, in North American red squirrels (*Tamiasciurus hudsonicus*), the effect of postnatal growth rate on juvenile survival did not depend on population density [26].

The effect of nestling mass on recruitment was also stronger for later-fledging sparrows. Food availability is often lower later in the season for temperate-breeding birds [50,51], though it is unknown how food resources vary seasonally on Kent Island. Higher-quality parents also usually breed earlier in the season; their offspring, which fledge earlier on average, may have higher recruitment probability due to improved early life care [30,31]. Older, more experienced parents, for instance, often breed earlier on Kent Island [45]. Later-fledging young also have less time to develop foraging and predator avoidance skills and to build up body reserves and moult before autumn migration [15,52]. Lighter fledglings may be less equipped to cope with these challenging late-season conditions and may have difficulty meeting minimum requirements to survive migration or may be forced to delay migration [52]. A previous study of bobolinks (*Dolichonyx oryzivorus*) and Savannah sparrows revealed that the effect of mass on juvenile survival depended on fledge date, with survival decreasing more strongly with fledge date for average-to-heavy nestlings and, contrary to the prediction, increasing with fledge date for lighter nestlings [25]. This result, however, may reflect higher levels of emigration of heavier fledglings rather than greater mortality [25]. In contrast, another study on bobolinks found that the effect of mass on juvenile survival did not depend on nest attempt number, a proxy of time of breeding and egg quality [53]. Other studies on passerines found no support for an interactive effect of timing of breeding with early life traits on annual juvenile survival [35,36].

Our findings of context-dependent effects of early life conditions on recruitment have important implications for life-history evolution. Based on our results, we would expect the strength of selection on nestling mass to fluctuate with population density, which varies substantially between years on Kent Island (figure 1b). When density is low and mass is less important for recruitment, parents may be able to maximize their fitness by producing more, lighter offspring [19]. When density is high, however, there will be more selection pressure to produce fewer, heavier offspring [19]. Similarly, selection on nestling mass should depend on timing of breeding. As breeding shifts earlier with climate change [54–56], selection on high nestling mass may weaken because of the reduced effect of mass on recruitment of early fledglings. As yet, pre-breeding temperatures on Kent Island have not shown a long-term increase with climate change, possibly due to increased fog formation in the Bay of Fundy or local effects of the cold Labrador Current, and we have, therefore, not observed an advance of nest initiation dates of Savannah sparrows since 1987 [57].

Failing to include context dependence in studies of the effects of early life conditions runs the risk of missing important patterns of variation in recruitment. The few studies that have evaluated interactions between indices of early life conditions, such as mass, and contextual factors including population density and timing of breeding have found important effects [11,25,26,58]. There may also be other important interactions between early life traits and environmental conditions. In red squirrels, for instance, the effect of food availability on over-winter survival of juveniles depended on litter size, with particularly low survival of juveniles born into large litters in low-food years [26]. Timing of breeding and population density may also interact: early breeding red squirrels had higher offspring survival than late breeders when population density was high, potentially because late-born offspring are less able to compete for territories [58], although we found no evidence for a similar effect here (population density × fledge date, electronic supplementary material, table S1). We suggest that future studies examining the effect of early life conditions on recruitment and survival of juveniles include context dependence.

A common limitation in studies of recruitment and juvenile survival is the difficulty of differentiating between mortality and permanent emigration from the study site, given high dispersal rates of juveniles [59,60]. Exact dispersal rates of juveniles in the study population are unknown, and if dispersal accounted for a large portion of individuals that did not return, the observed results could be related to dispersal as well as survival. For instance, lighter fledglings born in high-density years may perceive their own lower competitive ability for a territory in the following year and disperse farther. Lighter fledglings born later in the season may be less competitive on the wintering grounds, depart later on spring migration and be forced to disperse farther north to avoid phenological mismatch or because they are excluded from Kent Island due to late arrival [61,62]. However, for the following reasons, it is unlikely that a substantial proportion of juveniles disperse in the study population. First, because of high natal philopatry, most juveniles not resighted are likely to have died rather than dispersed. Second, few dispersed yearlings are found during censuses of surrounding areas (see §2). Finally, survival during the first 3 months post-fledging is low (approx. 0.2–0.3; [63]), which would require high survival during the migratory and overwintering periods if large numbers of juveniles disperse—unlikely, given that survival is generally low during migration [63,64]. While some individuals do disperse from the study site, observed patterns in return rates here are likely driven primarily by survival rather than by dispersal.

Our estimate of local recruitment, which averaged 12% across years, is among the lower published estimates of survival for songbird species, but not in the extreme [65]. One study of Savannah sparrows in agricultural habitats found a recruitment rate of approximately 0.2–0.3, though unlike our study, this rate includes immigration of yearling birds [66]. On Kent Island, a large proportion of juvenile mortality occurs in the post-fledging period [67], when inexperienced young fledglings are especially vulnerable to starvation and predation [7,34]. Post-fledging survival is frequently influenced by mass and fledge date [68], and mass may more strongly influence survival in the first few weeks post-fledging for late-fledging individuals than for early-fledging ones [34]. Thus, early life conditions may have the strongest influence on survival soon after juveniles leave the nest. Future work that investigates juvenile survival from fledging to departure for autumn migration will help us to better understand drivers of juvenile mortality.

## Data Availability

All data, metadata, and R code used to generate results are available from the Dryad Digital Repository [69]. Data also available at [70]. Supplementary material is available online [71].
